# Sauchinone alleviates dextran sulfate sodium-induced ulcerative colitis *via* NAD(P)H dehydrogenase [quinone] 1/NF-kB pathway and gut microbiota

**DOI:** 10.3389/fmicb.2022.1084257

**Published:** 2023-01-09

**Authors:** Kun Wu, Xianjun Liu, Xianglong Meng, Lingling Cao, Hao Li, Yingxin Bi, Mengyuan Wang, Mingchuan Wang, Yang Jiang

**Affiliations:** ^1^Department of Gastrointestinal Colorectal and Anal Surgery, The China-Japan Union Hospital of Jilin University, Changchun, China; ^2^College of Biological and Food Engineering, Jilin Engineering Normal University, Changchun, China; ^3^Department of Burns Surgery, The First Hospital of Jilin University, Changchun, China; ^4^School of Clinical Medical, Changchun University of Chinese Medicine, Changchun, China

**Keywords:** sauchinone, ulcerative colitis, NF-kB pathway, NQO1, gut microbiota

## Abstract

**Objective:**

This study evaluated the effects of sauchinone on dextran sulfate sodium (DSS)-induced ulcerative colitis (UC) mice model and investigated the underlying mechanisms of the downstream pathway and gut microbiota.

**Methods:**

The UC mice model was induced by DSS. The disease phenotypes were determined through pathological symptoms (body weight and disease activity index score), inflammation markers (histological and inflammatory factor detections), and colonic mucosal barrier damage (detection of tight junction proteins). The level of the NF-κB pathway was detected through marker proteins. Database and bioinformatics analyses were used to predict sauchinone-mediated downstream molecules that were previously identified by expression analysis. Mouse feces were collected to detect the V3–V4 region of the 16S rRNA gene.

**Results:**

In DSS-induced UC mice, sauchinone alleviated pathological symptoms, inhibited inflammation, and prevented mucosal barrier damage. Sauchinone further inhibited the NF-κB pathway by upregulating NAD (P) H dehydrogenase [quinone] 1 (NQO1) in DSS-induced UC mice. Moreover, sauchinone regulated the diversity and composition of the gut microbiota in mice, stimulating the growth of *Firmicutes* and inhibiting the growth of *Proteobacteria* and *Bacteroidetes*.

**Conclusion:**

Therefore, sauchinone exerted therapeutic effects on UC in mice by regulating the NQO1/NF-κB pathway and altering the gut microbiota. This provides a theoretical basis for developing sauchinone as a therapeutic agent and extends our understanding of its bioactivity.

## 1. Introduction

Ulcerative colitis (UC) is a complex, immune-mediated, and chronic inflammatory disease. Inflammation begins in the rectum involving any part of the colorectal mucosa, with classic presentations, such as bloody diarrhea, variable degrees of abdominal pain, and rectal emergencies (Ungaro et al., 2019). Current treatments for colitis include mesalamine, immunomodulators, corticosteroids, biologics, and small molecules (Kayal and Shah, 2019). Because UC is a chronic disease, the pathogenic factors are complex and variable, which results in lifelong treatments and an unclear course of pathogenesis.

**Figure 1:**
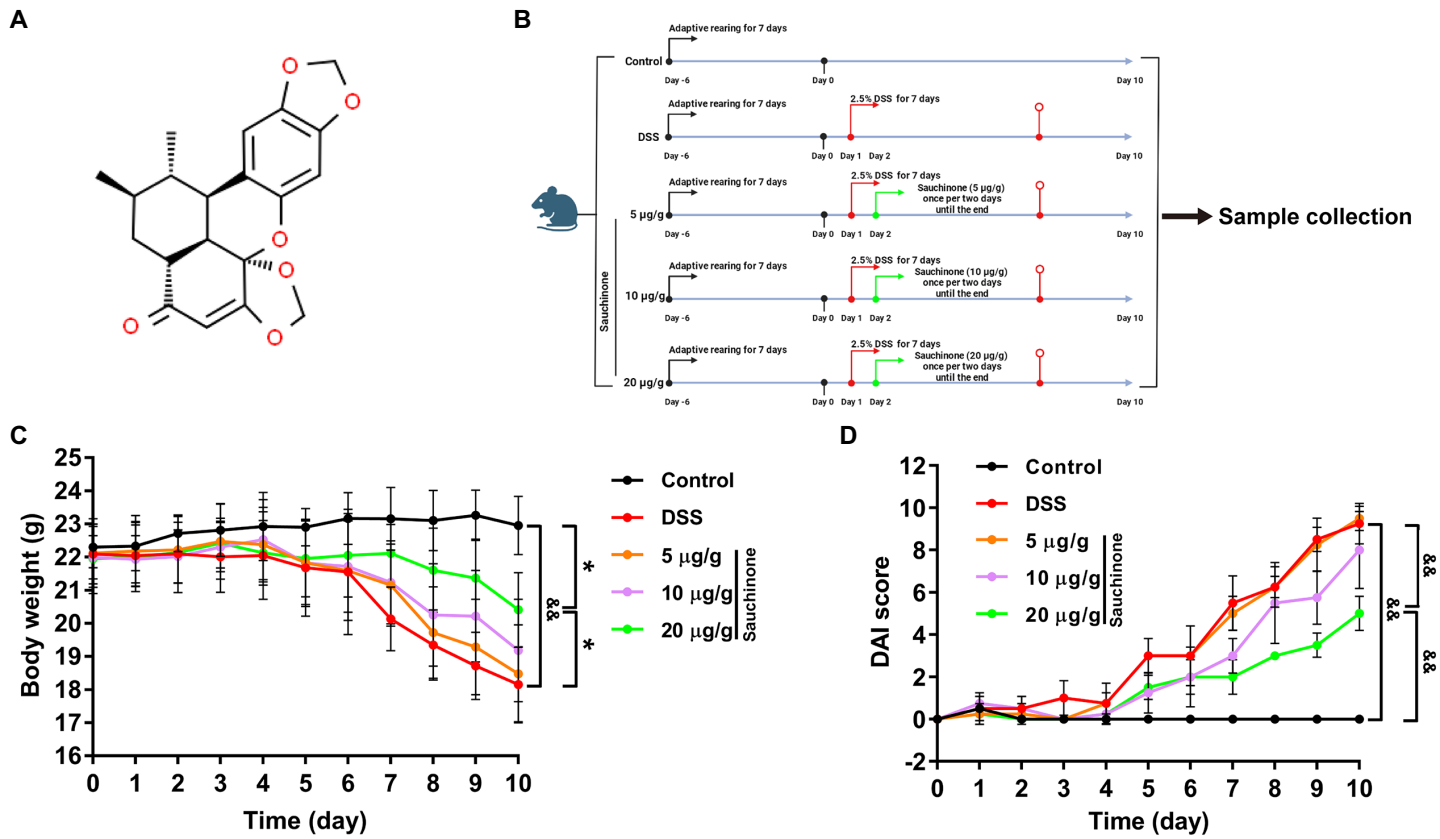
Effects of sauchinone on the pathological symptoms of dextran sulfate sodium (DSS)-induced UC mice. **(A)** The structural formula of sauchinone. **(B)** Schematic representation of the experimental design using the DSS-induced UC mice model and sauchinone treatment. **(C)** Body weight was measured. **(D)** Disease activity index (DAI) score was calculated. Data are expressed as mean ± standard deviation. ^*^p < 0.05 and ^&&^*p* < 0.01 vs. corresponding control; *n* = 4.

Natural product drugs have gradually attracted widespread attention for their health benefits, lack of side effects, and good therapeutic effects. Sauchinone (C_20_H_20_O_6_, Figure 1A) is a bioactive compound extracted from the root of *Saururus chinensis* (Saururaceae). The plant has been used in traditional Chinese medicine to treat various diseases. Increasing amounts of evidence have shown that sauchinone has antioxidant and anti-inflammatory properties, and it exerts protective effects in macrophages (Jeong et al., 2014), hepatocytes (Lee et al., 2014), and nerve cell (Song et al., 2003). Sauchinone primarily functions through regulating mitochondrial function (Kim et al., 2017), cell proliferation (Kim et al., 2021), and its involvement in molecular mechanisms, such as the TGF-β/Smad and AMPK pathways (Lee et al., 2014; Kim et al., 2017). Sauchinone reportedly has a therapeutic effect on UC and significantly ameliorates dextran sulfate sodium (DSS)-induced UC by regulating dendritic cells through Blimp-1 to inhibit inflammatory response (Xiu et al., 2020). However, the specific protective mechanisms remain unexplored.

Scientists have suggested that colitis is caused by altered epithelial barrier function and gut dysbiosis as characterized by compositional and diversity changes in the microbiota (Shen et al., 2018). Furthermore, researchers have increasingly wondered whether gut dysbiosis contributes to the immune impairment associated with UC, especially since gut microbiota is involved in the onset and development of UC. The composition and changes in the gut microbiota can be analyzed and characterized by high-throughput sequencing techniques, demonstrating the potential of this technology in advancing colitis treatment. The interaction between the intestinal microbes and the mucosal immune system has been identified as the key to chronic inflammation, and intestinal microbiome diversity may play a vital role in the pathogenesis of UC (Gao X. et al., 2018). Studies have shown that various drugs can affect the occurrence and development of UC by regulating the gut microbiota; however, whether this relationship applies to sauchinone is unclear.

In this study, we explored the role of the gut microbiota in regulating UC in response to sauchinone. We examined the specific mechanism by which sauchinone affects UC through the NF-κB pathway, clarifying its medicinal value and clinical effects.

## 2. Materials and methods

### 2.1. Chemicals and reagents

Dextran sulfate sodium (36,000–50,000 molecular weight), colitis grade, was provided by MP Biomedicals (Solon, OH, United States). Sauchinone was purchased from Med Chem Express Co., Ltd. (New Jersey, United States). Column tissue and cell protein extraction kit was provided by Epizyme Co., Ltd. (Shanghai, China). Primary antibodies in western blotting for Claudin-1, Occludin, ZO1, IL-6, TNF-α, NF-κB p65, p-IKB-α, and IKB-α were purchased from Huabio Co., Ltd. (Hangzhou, China). Primary antibodies for IL-1β, early growth response-1 (EGR1), and NAD(P)H dehydrogenase [quinone] 1 (NQO1) were supplied by ProteinTech Co., Ltd. (Chicago, IL, United States). Anti-p-p65 and secondary antibodies were provided by Abcam Co., Ltd. (Cambridge, United Kingdom). TRIzol for RNA extraction was obtained from Thermo Fisher Co., Ltd. (Waltham, United States). The EasyScript® All-in-One First-Strand cDNA Synthesis SuperMix and TransStart® Green qPCR SuperMix were purchased from Transgene Co., Ltd. (Beijing, China). BCA protein assay kit, BeyoECL Moon, and tissue RIPA lysis buffer were provided by Beyotime Co., Ltd. (Shanghai, China).

### 2.2. UC mice model and experimental design

Male C57BL/6 mice (6–8 weeks old, 20 ± 2 g, approval no. SCXK 2020–0001) were obtained from Shenyang Changsheng Company. All standards for animal feeding were implemented in accordance with the requirements of the ethics committee (approval no. 2021543) of the Changchun University of Chinese Medicine. The mice were randomly divided into five groups (*n* = 4/group) according to body weight after 1 week of adaptive rearing at temperatures of 18–23°C under a 12-h light/dark cycle. The five groups included the control, model, sauchinone low-dose (5 μg/g), middle-dose (10 μg/g), and high-dose groups (20 μg/g). Besides the control group, which was given distilled water, the other groups were continuously administered 2.5% (w/v) DSS drinking water for 7 days, which was replaced every morning to induce acute UC in the mice. To three treatment groups, sauchinone was administered intragastrically at different doses once per 2 days at 8: 00–10: 00 am until the end (Figure 1B, created with BioRender.com).

### 2.3. Evaluation of the disease activity index

Scores were calculated according to the disease activity index (DAI) scoring scale, combining weight loss, stool properties, and hematochezia scores. The scores for each factor were assigned as follows: weight loss (no change = 0; ≤ 5% = 1; 6–10% = 2; 10–15% = 3; and ≥ 16% = 4), stool properties (normal = 0; soft stool = 1; moderate diarrhea = 2; and diarrhea = 3), and hematochezia (no rectal bleeding = 0; slight rectal bleeding = 1; and bloodstains visible on stool = 2, rectal bleeding = 3). DAI = weight loss score + stool properties score + hematochezia score.

### 2.4. Sample collection

At the end of the treatment period, mice were euthanized by cervical dislocation, and colon tissue was collected for hematoxylin and eosin (H&E) staining and mRNA or protein expression analysis. During fecal collection for gut microbiota analysis, mice were individually isolated to collect fecal samples from each mouse separately.

### 2.5. H&E staining

Mouse colon tissues were fixed in a 10% neutral formalin solution. After fixation, paraffin-embedded 5 μm-thick sections were prepared, and H&E staining was performed for pathological observation and photography.

### 2.6. Real-time quantitative PCR

Quantitative PCR (qPCR) was used to assess mRNA expression. Total RNA was extracted from colon tissue by TRIzol reagent. Some samples were allocated for the direct detection of RNA concentration and quality, while the remaining RNA samples were stored at −80°C. According to the manufacturer’s instructions, mRNA was reverse-transcribed into cDNA using the kit, and mRNA expression was detected using the SYBR GREEN kit. *Actb* (β-actin) was used as the control to calculate the relative mRNA expression of the target genes using the 2^−∆∆Ct^ method. The primers used for qPCR are listed in Table 1. Each sample was run in triplicate.

**Table 1:** Sequences of primers for qPCR.

	Forward (5’→3’)	Reverse (5’→3’)
TNF-α	GGGCCATAGAACTGATGAGAGGG	TCCAGAACTCCAGGCGGT
IL-6	TTGTATGAACAACGATGATGCACT	TGTTCTTCATGTACTCCAGGTAGC
IL-1β	GTAGTGCAGTTGTCTAATGGGAACG	GCTACCTGTGTCTTTCCCGTGGA
ZO-1	CACTACAGTATGACCATCCTCA	TTCTCTGTTCACACAGATAAGC
Occludin	TTATACTCCTGCAGACCTGCATCAA	GACGGACCCTGACCACTATGAAACA
Claudin-1	TCATCTTCCAGGCACCTCATGCA	GTGCAAAGTCTTCGACTCCTTGCTG
NQO1	ACAATATCTGGGCTCAGGCGT	GGGAGGTACTCGAATCTGACCTCT
EGR1	AGCCGAGCGAACAACCCTATG	AGGCAACCGAGTCGTTTGGCT
GDF15	CGGTTGACGCGGAGTAGCAGCT	GCTGCATGCCAACCAGAGCCG
TNFSF4	CAATCCAAAGACTCAGAGGAGCAG	GCCCATCGCACTTGATGACAAC
UBD	ATTCAGCCTCTGACTACAGACATG	CACTTTGTCATTCTCAGTGGTCT
ACTB	TACCACCAGACAGCACTGTGTT	GAGGCTCTTTTCCAGCCTTCCTT

### 2.7. Western blotting

Western blotting was used to assess protein expression. Total protein was extracted using the column tissue and cell protein extraction kit. Afterward, the protein concentration was detected with a BCA kit, and the protein samples were boiled. Each sample separated by sodium dodecyl sulfate-polyacrylamide gel was a pool of equal protein samples from four mice of each group. The process was stopped when bromophenol blue migrated to the bottom of the gel. The proteins were transferred from the gel to the polyvinylidene difluoride membrane by the wet transfer method. After blocking, primary and secondary antibodies were incubated with the proteins on the membrane. Lastly, protein detection was performed on a developer (Weltech) using an ECL kit. The protein bands were calculated using the ImageJ software. β-actin was used as the control. Data from three independent experiments were analyzed.

### 2.8. Prediction of molecules targeted by sauchinone

This prediction was performed by taking the intersection between the differentially expressed genes (DEGs) of a RNA sequencing dataset, generated from sauchinone-treated HepG2 cells (Chae et al., 2018), and a dataset of NF-κB pathway-related genes. The pathway-related genes were acquired from the PathCards database (pathcards.genecards.org/; Belinky et al., 2015). The results are displayed in a Venn diagram.

### 2.9. 16S rRNA sequencing analysis

To identify microbes within the gut of mice with induced UC, feces were sampled for 16S rRNA gene sequencing. Fecal bacterial DNA was extracted using a Rapid DNA SPIN extraction kit. The DNA extraction quality was assessed using 0.8% agarose gel electrophoresis. The sequences of primers used to amplify the V3–V4 region of the 16S rRNA gene were as follows: forward primer (5-ACTCCTACGGGAGGCAGCA-3) and reverse primer (5-GGACTACHVGGGTWTCTAAT-3). The 16S rRNA sequencing of fecal bacteria was performed using the Illumina NovaSeq platform (Shanghai Personalbio Biotechnology Co., Ltd., Shanghai, China). Microbiome bioinformatics analysis was performed using QIIME 2 (2019.4) according to official tutorials,^1^ with slight modifications. Raw sequence data were demultiplexed using the Demux plug-in. Sequences were then quality-filtered, denoised, merged, and purged of chimeras using the DADA2 plug-in. For the bacterial community in each sample, the α-diversity and β-diversity indices were estimated using the diversity plug-in and according to the amplicon sequence variants (ASV) distribution. Bioinformatics analysis was performed using the Personalbio platform.^2^

### 2.10. Statistical analysis

Quantified data are expressed as the mean ± SD. The data were assessed for normality using the Shapiro–Wilk test and analyzed by one-way ANOVA followed by *post-hoc* Dunnett’s or Sidak test using GraphPad Prism (version 7.04) statistical package. *p* < 0.05 was considered to indicate a statistically significant difference.

## 3. Results

### 3.1. Effects of sauchinone on the pathological symptoms of DSS-induced UC mice

In DSS-induced UC mice, we found that the degree of weight loss was reduced after sauchinone treatment (Figure 1C). Although weight had been lost for model and sauchinone-treated groups alike, the model group lost weight more rapidly. Additionally, the model group exhibited diarrhea and hematocheziac after induction by DSS in comparison to the control group. The DAI score (Figure 1D) in the model group was significantly higher than that in the control group, and the score was lower for groups that received sauchinone. These results indicate that sauchinone could significantly improve DSS-induced UC symptoms in mice and alleviate colon damage. Sauchinone had a significant therapeutic effect on DSS-induced UC in mice.

### 3.2. Effects of sauchinone on inflammation in DSS-induced UC mice

**Figure 2:**
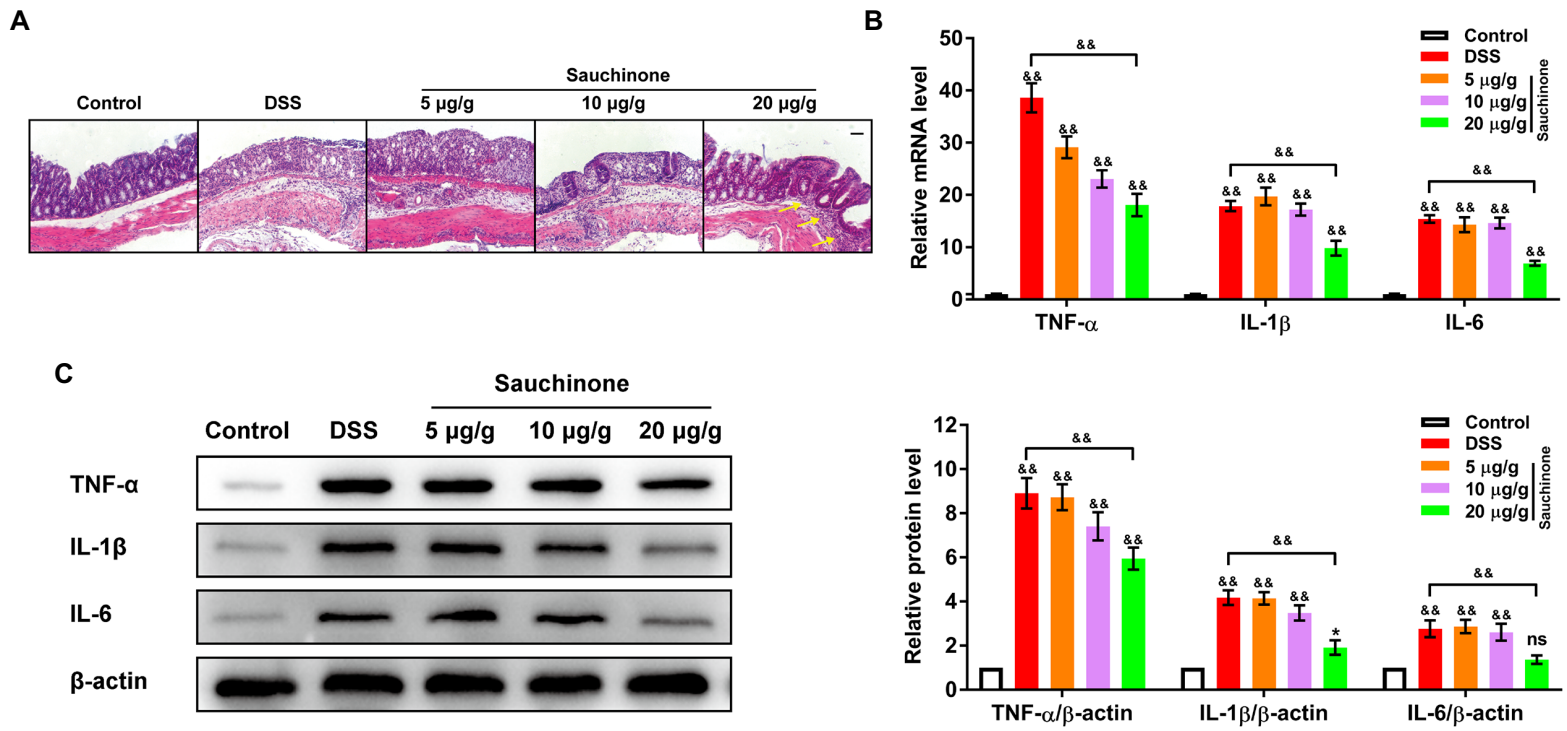
Effects of sauchinone on inflammation in DSS-induced UC mice. **(A)** Representative H&E-stained colon tissue sections (yellow arrow indicates inflammatory cell infiltration; scale bar = 50 μm). The expression levels of inflammatory cytokines (TNF-а, IL-1β, and IL-6) in colon tissues were detected by **(B)** qPCR and **(C)** western blot. Data are expressed as mean ± standard deviation. ^*^p < 0.05 and ^&&^*p* < 0.01 vs. corresponding control, ns indicates no significant difference; *n* = 4.

We further evaluated the effects of sauchinone on colon inflammation by measuring inflammation-associated biomarkers. Figure 2A shows the H&E staining results of the colon tissues. In the control group, the colonic mucosa, crypts, and submucosa were intact. Additionally, the tissues were less infiltrated by inflammatory cells, and there was no inflammatory response. Conversely, significant tissue damage and inflammation, indicated by mucosal erosion and infiltration of inflammatory cells, were observed in the colon samples of the model group. Sauchinone treatment improved colon condition compared to the model group. Sauchinone treatment (20 μg/g) significantly alleviated colitis symptoms, resulting in normal crypt morphology, increased numbers of goblet cells and epithelial cells, and a low number of infiltrating inflammatory cells that is comparable to observations in the submucosa of control tissues. Next, we measured the levels of the related inflammatory cytokines (TNF-α, IL-1β, and IL-6). Compared with the model group, high-dose sauchinone significantly reduced the levels of inflammatory factors. As shown in Figure 2B, compared with the control group, the mRNA expression levels of TNF-α, IL-1β, and IL-6 significantly increased after DSS inducement. In contrast, sauchinone significantly decreased mRNA expression levels of inflammatory factors. The protein expression trends were consistent with the qPCR results (Figure 2C). These results revealed that sauchinone suppressed inflammation in the DSS-induced UC mice model.

### 3.3. Effects of sauchinone on colonic mucosal barrier damage in DSS-induced UC mice

**Figure 3:**
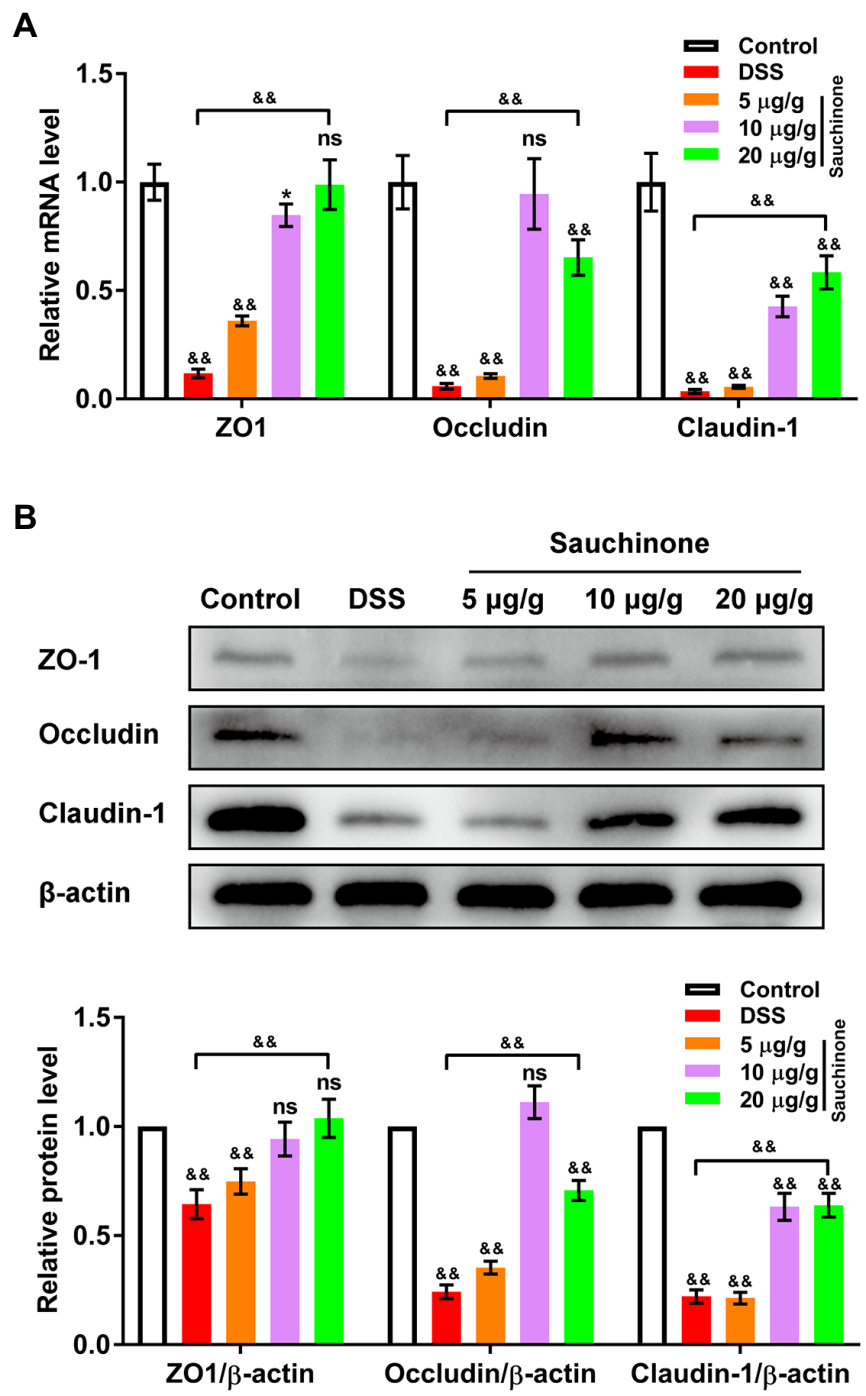
Effects of sauchinone on colonic mucosal barrier damage in DSS-induced UC mice. The expression levels of tight junction proteins (ZO-1, occludin, and claudin-1) were detected by **(A)** qPCR and **(B)** western blot. Data are expressed as mean ± standard deviation. ^*^p < 0.05 and ^&&^*p* < 0.01 vs. corresponding control, ns indicates no significant difference; *n* = 4.

To assess for the protective effects of sauchinone on the mucosal barrier of the colon, we measured the expression levels of biomarkers (tight junction proteins ZO-1, Occludin, and Claudin-1) related to mucosal barrier damage. As shown in Figure 3A, qPCR revealed that marker mRNA levels in the model group were significantly lower than those in the control group. Compared with the model group, sauchinone treatment (20 μg/g) significantly reversed the downregulation caused by DSS. Trends in protein levels from western blotting analysis were consistent with those of corresponding mRNA from qPCR (Figure 3B). Based on its apparent effectiveness, 20 μg/g sauchinone was used for subsequent experiments. These data suggest that sauchinone could maintain the integrity of the colonic mucosal barrier through the expression of junction proteins.

### 3.4. Sauchinone regulated the NQO1/NF-κB pathway in DSS-induced UC mice

**Figure 4:**
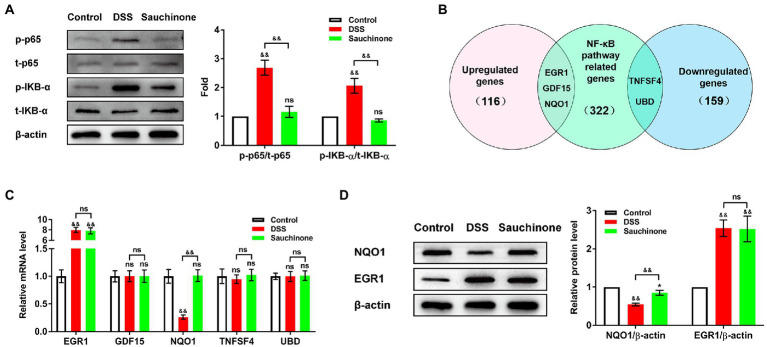
Sauchinone regulated the NQO1/NF-κB pathway in DSS-induced UC mice. **(A)** The levels of NF-κB pathway-associated proteins (p-p65, t-p65, p-IKB-α, and t-IKB-α) were detected by western blot. **(B)** Predicted target molecules of sauchinone (EGR1, GDF15, NQO1, TNFSF4, and UBD) are displayed in a Venn diagram. **(C)** *Egr1, Gdf15, Nqo1, Tnfsf4*, and *Ubd* expression levels were detected by qPCR. **(D)** NQO1 and EGR1 expression levels were detected by western blot. Data are expressed as mean ± standard deviation. ^*^p < 0.05 and ^&&^*p* < 0.01 vs. corresponding control, ns indicates no significant difference; *n* = 4.

We further investigated the downstream mechanisms of sauchinone-mediated regulation. First, we verified the effects of sauchinone on the NF-κB pathway in the DSS-induced UC mice model by testing the expression levels of pathway-associated proteins (p-p65, t-p65, p-IKB-α, and t-IKB-α). As shown in Figure 4A, western blotting results revealed that the ratios of p-p65/t-p65 and p-IKB-α/t-IKB-α increased in the model group compared with the control group. On the other hand, these ratios decreased in the sauchinone-treated group compared to the model group. This suggests that sauchinone inhibited the NF-κB pathway in the DSS-induced UC model. To elucidate how sauchinone mediates this effect, we investigated potential target molecules of sauchinone. We performed a preliminary prediction of candidates in the mouse colon by selecting the DEGs from previously published RNA sequencing data on sauchinone-treated HepG2 cells (116 upregulated DEGs and 159 downregulated DEGs). We further compared these DEGs to NF-κB pathway-related genes (322 genes) from a PathCards dataset. Focusing on the intersection between these datasets, we selected *Egr1*, growth differentiation factor 15 (*Gdf15*), *Nqo1*, TNF superfamily 4 (*Tnfsf4*), and ubiquitin D (*Ubd*) to be candidate genes that encode molecules involved in sauchinone-induced inhibition of the NF-κB pathway (Figure 4B). We speculated that EGR1, GDF15, and NQO1 would be upregulated by sauchinone, that changes in their expression would relate to the regulation of the NF-κB pathway, and that the opposite would happen for TNFSF4 and UBD. Further screening was performed using expression analysis. As shown in Figure 4C, qPCR showed that *Nqo1* expression decreased in the model group compared with the controls. This downregulation was reversed in the sauchinone-treated group. *Egr1* expression increased in the model group compared with the control group and did not significantly change in the sauchinone-treated group. None of the other three molecules changed across all groups. We performed western blotting to verify NQO1 and EGR1 protein expression levels in colon tissues, and the results were consistent with those of qPCR (Figure 4D). These data demonstrate that sauchinone regulated inflammation in DSS-induced UC *via* the NQO1/ NF-κB pathway.

### 3.5. Sauchinone modulated the composition and structure of gut microbiota in DSS-induced UC mice

**Figure 5:**
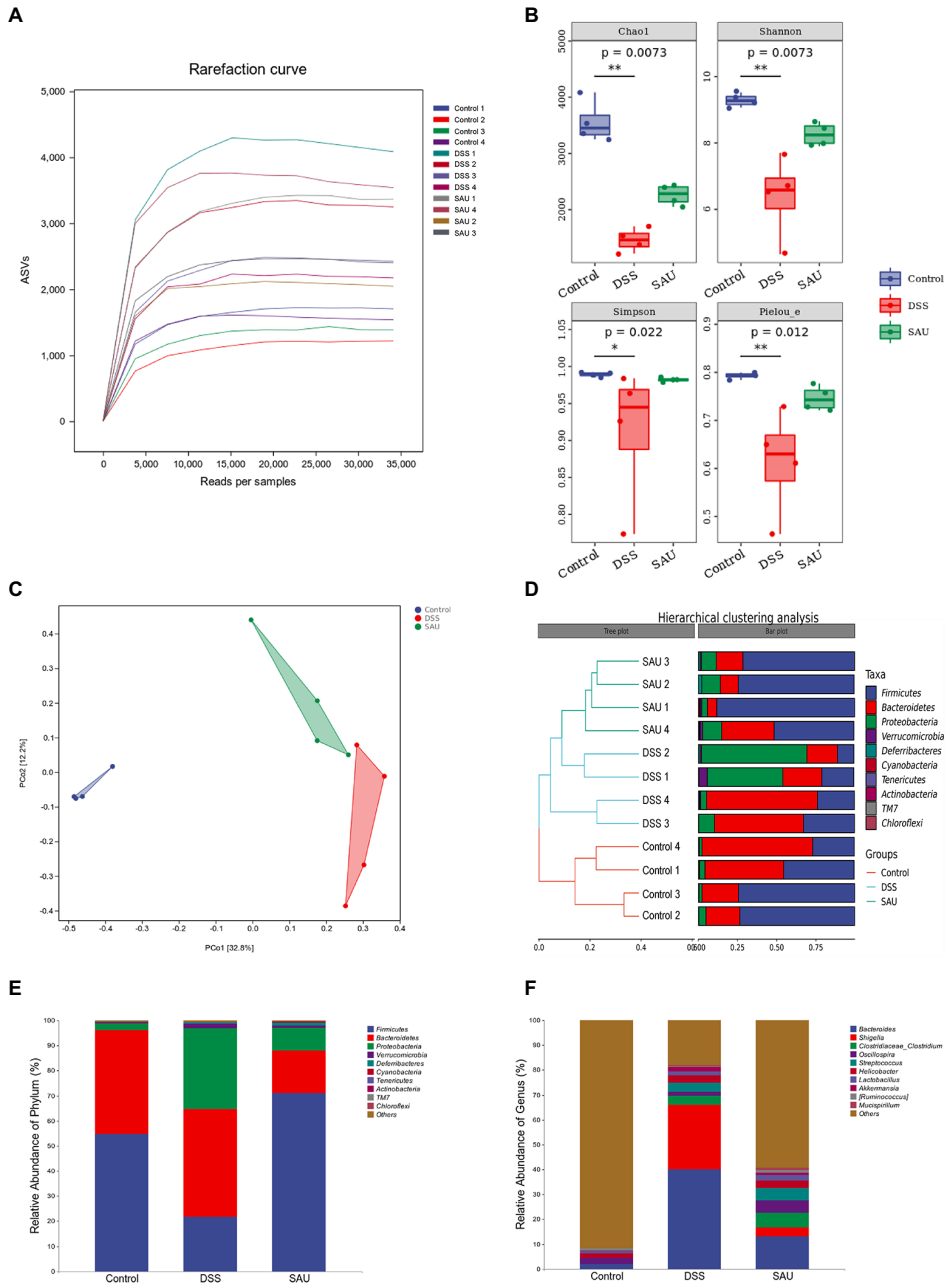
Effects of sauchinone on the composition and structure of gut microbiota in DSS-induced UC mice. **(A)** The rarefaction curves. **(B)** α-diversity based on the Shannon, Chao 1, Simpson, and Pielou indices. **(C)** Bray-Curtis-based PCoA plot based on the ASV level (*p* = 0.001). **(D)** Hierarchical clustering analysis at the phylum level. **(E,F)** Average taxonomic profile analysis at the phylum and genus level.

Given that gut microbiota plays a critical role in regulating UC development, we investigated the effect of sauchinone on the composition and structure of gut microbiota in DSS-induced UC mice. The original reads obtained by 16S rRNA sequencing were subjected to de-priming, quality filtering, de-noising, merging, and de-chimera steps to obtain a total of 534,272 singleton-removed sequences, with an average of 44,522 sequences per sample. The rarefaction curve (Figure 5A) indicated that the number of ASVs increased significantly with sequencing time. However, the rise in the number of ASVs gradually slowed, indicating that the current amount of sequencing is sufficient to detect most species and reflect the variety in microorganisms from different samples. α-diversity indices (including Chao 1, Shannon, Simpson, and Pielou) were analyzed. Sauchinone administration mitigated the significantly decreasing indices in the DSS-induced UC mice group (Figure 5B). This indicates that sauchinone affected the community diversity and richness of the gut microbiota in UC mice. Based on the number of ASVs, principal co-ordinate analysis (PCoA) evaluation and hierarchical clustering analysis were performed to test the composition and structure of gut microbiota among the three treatment groups. We found that the flora structure of the control group was markedly separated from that of the other two groups. The model group was somewhat distanced from the sauchinone group, consistent with the phylogenetic tree results. This suggests that sauchinone could modulate the community structure of the gut microbiota in UC mice (Figures 5C,D). To investigate the regulatory effect of sauchinone on gut microbiota composition, we analyzed the relative abundance of microorganisms at the phylum and genus levels. At the phylum level, the relative abundance of *Firmicutes* in the DSS group was lower than that in the control group; this lowered abundance was reversed by sauchinone. Additionally, the relative abundances of *Bacteroidetes*, *Proteobacteria*, and *Verrucomicrobia* in the DSS group were higher than those in the control group, while sauchinone reduced these abundances (Figure 5E). At the genus level, the relative abundances of *g_Bacteroides* and *g_Helicobacter* in the DSS group were higher than those in the control group; meanwhile, sauchinone reduced these abundances. Furthermore, the relative abundances of *g_Oscillospira* and *g_Ruminococcus* in the DSS group were lower than those in the control group; this difference was reversed by sauchinone. Lastly, between the DSS and sauchinone groups, the relative abundances of *g_Shigella*, *g_Streptococcus*, and *g_Akkermansia* differed; however, they did not rank in the top 10 abundance in the control group (Figure 5F).

### 3.6. Effect of sauchinone on the linear discriminant analysis effect size analysis of gut microbiota in DSS-induced UC mice

**Figure 6:**
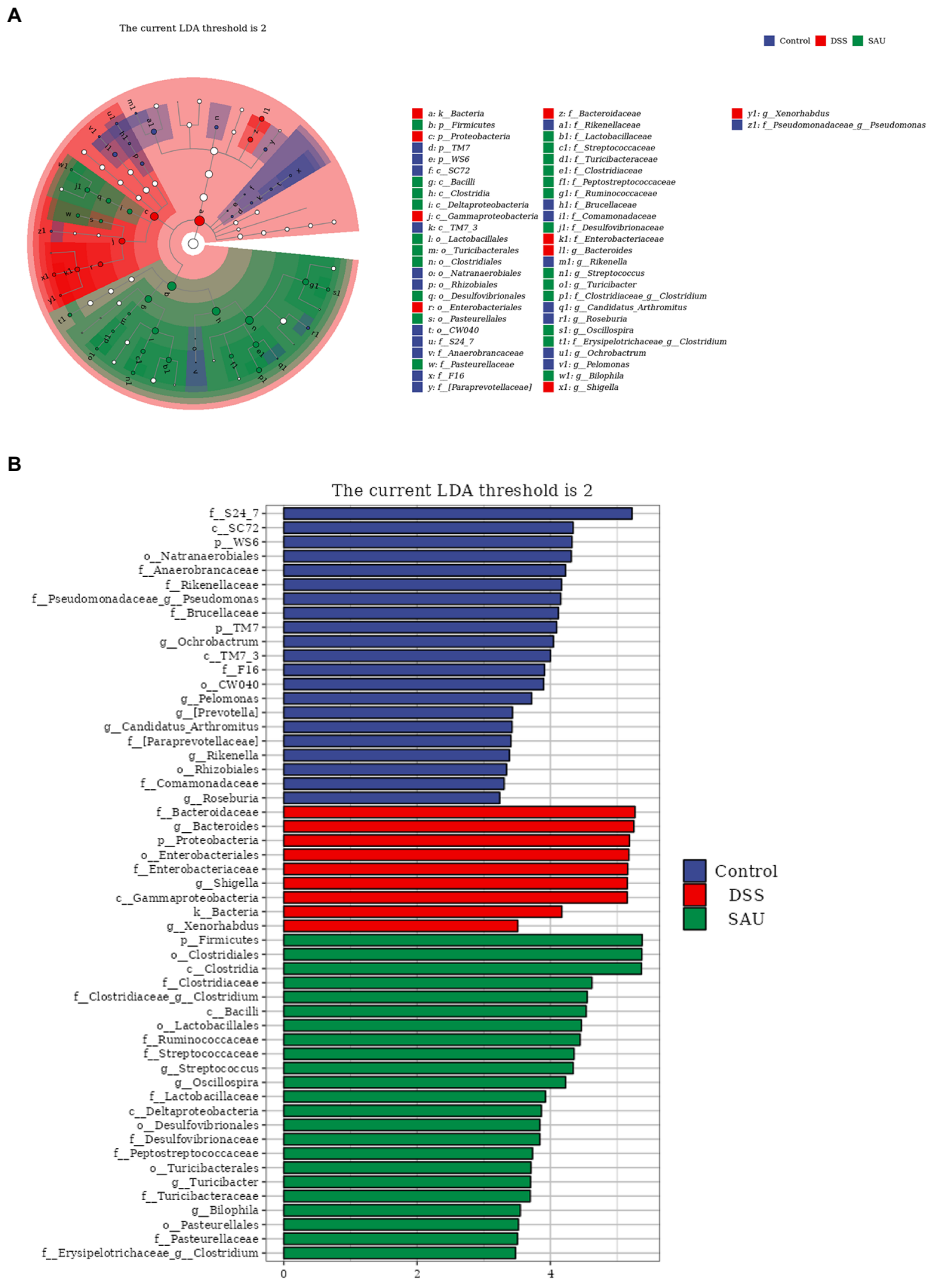
Linear discriminant analysis effect size revealed differences in taxa abundances. **(A)** The cladogram of detected taxa. **(B)** Taxa with significant differences based on linear discriminant analysis effect size with a threshold score of 2 and *p* < 0.05.

The linear discriminant analysis (LDA) effect size (LEfSe) was used to analyze the microbial features. The resultant score showed that the abundance of *Bacteroidaceae*, *Bacteroidetes*, *Proteobacteria*, *Shigella*, and *Enterobacteriaceae* increased in the DSS group. Meanwhile, *Firmicutes*, *Clostridia*, and *Clostridiales* had higher scores in the sauchinone group (Figures 6A,B), suggesting that sauchinone alleviates inflammation related flora.

## 4. Discussion

Currently, treatment options for UC depend on the extent, severity, and course of the disease. Mesalamine is the first-line treatment for UC. In severe cases, oral mesalamine or a combination of mesalamine and glucocorticoids are viable options as well. Severe cases require intravenous corticosteroid or adjuvant therapy with immunosuppressants and TNF-α monoclonal antibodies, and more severe cases require surgery (Kayal and Shah, 2019; Ungaro et al., 2019).

Bioactive compounds have been reported in the treatment of UC as well; they include flavonoids, acids, polysaccharides, terpenoids, phenols, alkaloids, quinones, and bile acids (Cao et al., 2019). Luteolin, a natural flavonoid compound, significantly reduced colon damage in UC rats and suppressed colon inflammation by reducing TNF-α and IL-6 secretion (Chen et al., 2007). It also decreased levels of NF-κB, IL-17, and IL-23, as well as increased the expression of PPAR-γ in colon tissue (Li et al., 2021). Gallic acid, another bioactive compound, has anti-inflammatory properties. In UC, gallic acid plays a potent anti-inflammatory role by significantly reducing IL-21 and IL-23 expression, as well as the suppression of NF-!B p65 and IL-6/p-STAT3^Y705^ activation (Pandurangan et al., 2015a,b). Studies show that andrographolide reduced intestinal damage by inhibiting the expression of p-p65, p-IκBα, and COX-2 and increasing the expression of PPAR-γ. Andrographolide derivative also inhibited the activation of NF-κB and MAPK pathways, decreased TNF-α and IL-6 levels, and reduced myeloperoxidase activity in UC mice (Yang et al., 2016; Gao Z. et al., 2018). Curcumin, a plant polyphenol derived from turmeric, has also been shown to modulate the NF-κB pathway, downregulate the expression of inflammatory cytokines like IL-1, IL-6, IL-8, and TNF-α (Wang et al., 2018), and block the infiltration of inflammatory cells such as CD4^+^ and CD8^+^ T cells (Deguchi et al., 2007).

Our study showed that sauchinone has the potential to treat UC, consistent with results reported by Guo et al. In TNBS-induced colitis mice, they found that sauchinone could improve the inflammatory CD4^+^ T cells response in the mucosa and peripheral blood. Additionally, they found that sauchinone significantly inhibited Th17 cell differentiation but promoted IL10 production (Xiao et al., 2020). Moreover, sauchinone had been reported to significantly inhibit miR-340 expression in Th17 cells (Chen et al., 2020). In dendritic cells, Blimp1 knockout eliminated sauchinone-mediated inhibition of pro-inflammatory cytokine and promoted the production of immunomodulators. This indicated that sauchinone significantly improved DSS-induced UC by modulating dendritic cells through Blimp-1 to suppress the inflammatory response (Xiu et al., 2020). In this study, we observed that sauchinone inhibited the upregulating expression of TNF-α, IL-1β, and IL-6 in DSS-induced UC mice. Moreover, we found that sauchinone reversed colonic mucosal barrier damage by verifying the restoration of tight junction protein expression. These data further reinforced the antagonistic roles of sauchinone against UC and contributed to the elucidation of mechanisms by which the roles are fulfilled.

Sauchinone is a structurally unique lignin isolated exclusively from *Saururus chinensis*. Sauchinone has anti-inflammatory, antioxidant, anticancer, neuroprotective, hepatoprotective, and lipid metabolism effects. Sauchinone has potent anti-inflammatory effects on hyperglycose-induced vascular endothelial cell through the Nrf-2 pathway-dependent HO-1 expression (Li et al., 2014). In hepatoprotective roles, sauchinone significantly reduced CCl_4_-induced liver fibrosis and TGF-β1-induced activation of hepatic stellate cells (Lee et al., 2014). Sauchinone further reduced hepatic steatosis by modulating lipid metabolism PPAR signaling pathway, downregulating liver subtilisin/kexin type 9 expression, activating liver low density lipoprotein LDL receptor expression, and increasing LDL-cholesterol uptake in obese mice (Chae et al., 2018).

In our study, we found that sauchinone modulated the expression of NF-κB pathway components. The NF-κB signaling is essential to inflammatory and immune responses. In other studies, sauchinone was found to reduce the expression of TNF-α by inhibiting the activation of NF-κB pathways and the generation of osteoclasts, and plays an anti-inflammatory role in treating inflammatory bone lytic diseases (Han et al., 2007). Meanwhile, sauchinone suppresses the inflammatory response in IL-1𝛽-stimulated human chondrocytes by inhibiting the expression of NF-κB signaling pathway components (Gao Y. et al., 2018), thereby reducing osteoarthritis (Wu et al., 2018). Our study demonstrated that sauchinone could reverse the activation of the NF-κB pathway in the DSS-induced UC model, suggesting that sauchinone exerts a wide range of inhibitory effects on the NF-κB pathway.

To predict regulators downstream of sauchinone, we examined genes involved in the NF-κB pathway whose expression levels were likely to be influenced by sauchinone based on a previous study (Chae et al., 2018). We then verified and evaluated how sauchinone would impact the expression of these candidate genes in DSS-induced UC mice. Our study predicted five potential gene products, involved in the NF-κB pathway, that might have been targets of sauchinone (EGR1, GDF15, NOQ1, TNFSF4, and UBD). NQO1 is a widely distributed, highly inducible enzyme with antioxidant, antitumor, and cytoprotective effects (Dinkova-Kostova and Talalay, 2010). NQO1 is highly expressed in tumor cells, and its silencing increases NF-κB levels and upregulates transcription associated with inflammation and tumorigenesis (Thapa et al., 2014). Upregulation of NQO1 expression inhibits NF-κB expression levels, thereby attenuating the inflammatory response (Lim and Song, 2021). In the context of UC, one study found that UC in mice could be prevented by upregulating NQO1 and downregulating NF-κB-related proteins (Zhang et al., 2021). Our results agree with this conclusion; furthermore, we demonstrated for the first time that sauchinone alleviates DSS-induced UC *via* the NQO1/NF-κB pathway. This process may be related to the anti-inflammatory effects of NQO1. In addition to NQO1, we also included EGR1 among our candidate molecules. The transcription factor EGR1 plays an important role in inflammation and immune-related gene regulation (McMahon and Monroe, 1996). EGR1 expression was significantly increased in the colonic mucosa in UC mice model (Tolstanova et al., 2012), consistent with our results. However, we found that EGR1 expression was not under sauchinone regulation, indicating it was not involved in sauchinone-mediated inhibition of the NF-κB pathway in the DSS-induced UC model. Furthermore, we found that the expression of GDF15, TNFSF4, and UBD was unaffected by sauchinone administration. GDF15, a peptide hormone in the TGF-β family, exerts a protective effect when overexpressed in tissues under pathological conditions (Assadi and Zahabi, 2020). It is overexpressed in the late stages of malignancy and is a potential therapeutic target for the treatment or prevention of cancer (Lerner et al., 2016). TNFSF4 is a cytokine in the tumor necrosis factor ligand family and is expressed in activated antigen-presenting cells and vascular endothelial cells. TNFSF4 increases the risk of myocardial infarction (Wang et al., 2005) and systemic lupus erythematosus (Cunninghame Graham et al., 2008) in humans. Research shows a reduction in *TNFSF4* DNA methylation in UC (Cooke et al., 2012). UBD, a member of the ubiquitin-like modifier family, is regarded as a new marker of human regulatory T cells (Ocklenburg et al., 2006). As one essential regulator of NF-κB (Wagner et al., 2008), its overexpression inhibits NF-κB activation. Furthermore, UBD-mediated regulation of NF-κB may be targeted for new cancer therapeutics. UBD expression was also reportedly downregulated in the colonic mucosa of patients with UC, suggesting its role in the pathogenesis of colonic inflammation (Camarillo et al., 2020). Our results, however, showed that these three proteins did not change across all groups, which may owe to differences between species. In summary, our study has expanded the understanding of possible mechanisms associated with sauchinone-mediated bioactivity. However, further research concerning the underlying mechanisms is required.

An additional factor mediating the protective effects of sauchinone is the gut microbiota, which colonizes the gut mucosa and is involved in human physiological metabolism, immune regulation, and homeostasis. Though the etiology of UC is still unclear, several studies have shown that UC pathogenesis associates with gut microbiota disorders and intestinal mucosal barrier dysfunction, suggesting that changes in the structure and abundance of gut microbiota play an important role in the development of UC (Gao X. et al., 2018; Shen et al., 2018). In the intestinal mucosa of patients with UC, the presence of large amounts of activated NF-κB and pro-inflammatory cytokines (IL-1β, IL-6, IL-12, TNF-α, etc.), in combination with bacteria such as *Clostridium*, *Streptococcus*, *Bacteroides*, *Veillonella*, and *Escherichia coli*, can produce pro-inflammatory factors that indirectly reinforce NF-κB pathway activation under specific conditions. This reduces the amount of apoptosis in inflammatory and immunologically active cells, producing a persistent local inflammatory response (Vieira-Silva et al., 2019; Zhang et al., 2019). Consequently, alterations in the structure of the gut microbiota are important factors in UC pathogenesis. In this study, we found that the abundance of *Firmicutes* and *Cyanobacteria* in the model group decreased significantly. In contrast, the abundance of *Proteobacteria* and *Verrucomicrobia* in the DSS-induced group increased significantly. Sauchinone treatment reversed the changes in relative abundance of these flora. These results suggest that sauchinone can remodel murine gut microbiota structure, leading to the alleviation of UC. *Firmicutes* include gram-positive bacteria that play a key role in host nutrition and metabolism through the synthesis of short-chain fatty acids. They regulate hunger and satiety through metabolites that are indirectly linked to functions in other tissues and organs (Stojanov and Berlec, 2020). Supplementation with *Firmicutes* can prevent or treat UC by downregulating colonic inflammation and the Th17 pathway in UC mice (Natividad et al., 2015). *Bacteroidetes* are gram-negative bacteria associated with immune regulation. They interact with cellular receptors and enhance immune responses through cytokine synthesis (Zhou and Zhi, 2016). *Proteobacteria* is a proliferates in the intestine when the organism is in a state of chronic or acute inflammation, causing a biological imbalance (Shin et al., 2015). In children with severe UC, the gut microbiota exhibited a 5% decrease in the relative signal of *Firmicutes* and a 3.6% increase in *Proteobacteria*, suggesting a strong relationship between *Proteobacteria* and UC (Michail et al., 2012). *Firmicutes*, *Bacteroidetes*, and *Proteobacteria* are the major differentially abundant bacteria in UC, the alterations of which we have shown to be consistent with observed trends in other studies. Notably, *Bacteroidetes* increased in abundance in our UC model mice, though this was abrogated by sauchinone treatment. This could have been due to factors, such as raising conditions, modeling strategy, and mouse source. Overall, our study revealed that sauchinone regulated the diversity and composition of the gut microbiota in DSS-induced UC mice.

## 5. Conclusion

**Figure 7:**
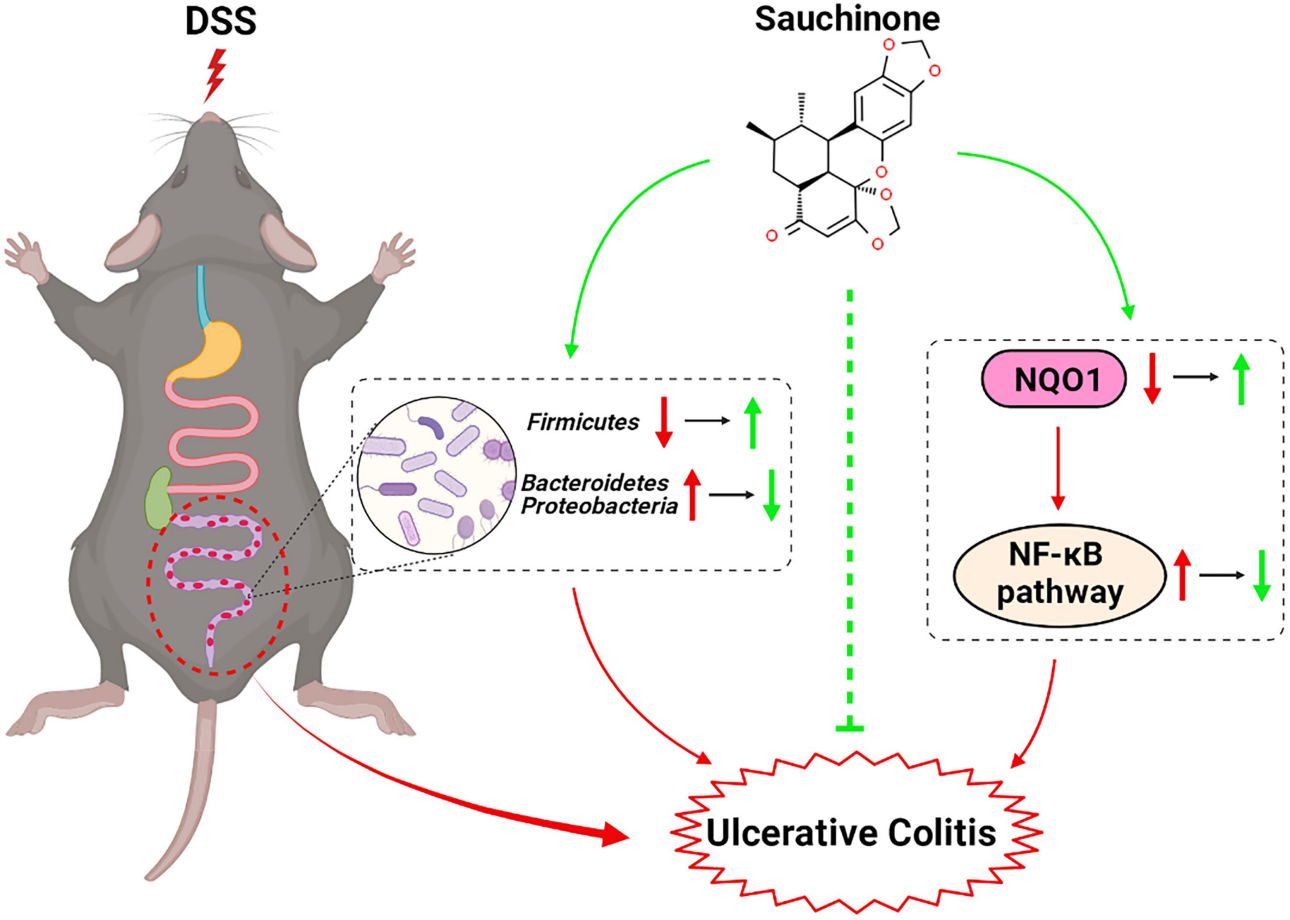
Schematic for the effects and mechanisms of sauchinone bioactivity in DSS-induced UC mice.

Sauchinone improved pathological symptoms, alleviated inflammation, and prevented mucosal barrier damage in DSS-induced UC mice. We found that the NQO1/NF-κB pathway and altered gut microbiota were associated with these effects (Figure 7, created with BioRender.com). These findings extended our understanding of the role and mechanisms of sauchinone bioactivity in UC, supporting further investigations into sauchinone as a novel therapeutic agent for UC treatment.

## Data availability statement

The datasets presented in this study can be found in online repositories. The names of the repository/repositories and accession number(s) can be found at: https://www.ncbi.nlm.nih.gov/, PRJNA894308.

## Ethics statement

The animal study was reviewed and approved by the ethics committee of Changchun University of Chinese medicine (approval no. 2021543).

## Author contributions

YJ and XL conceptualized and designed the study and revised the manuscript, contributed to funding acquisition, and guided and supervised the whole study. KW, XL, XM, LC, HL, YB, and MengyuanW performed the experiments. KW, XL, and MengyuanW contributed to results analysis. KW completed the first draft of the manuscript. KW, XL, and YJ confirm the authenticity of all the raw data. All authors contributed to the article and approved the submitted version.

## Funding

This study was supported by the Department of Science and Technology of Jilin Province (No. 20200201430JC and 20220202076NC), Jilin Province Development and Reform Commission (No. 2021C043-9), Education Department of Jilin Province (No. JJKH20220194KJ), and the PhD Research Project of Jilin Engineering Normal University (No. BSKJ201923).

## Conflict of interest

The authors declare that the research was conducted in the absence of any commercial or financial relationships that could be construed as a potential conflict of interest.

## Publisher’s note

All claims expressed in this article are solely those of the authors and do not necessarily represent those of their affiliated organizations, or those of the publisher, the editors and the reviewers. Any product that may be evaluated in this article, or claim that may be made by its manufacturer, is not guaranteed or endorsed by the publisher.

## Footnotes

^1^ https://docs.qiime2.org/2019.4/tutorials/

^2^ https://www.genescloud.cn/home

## Abbreviations

UC: Ulcerative colitis
DSS: Dextran sodium sulfate
DAI: Disease activity index
H&E: Hematoxylin and eosin
qPCR: Quantitative PCR
ASV: Amplicon sequence variants
DEG: Differentially-expressed genes
EGR1 and *Egr1*: Early growth response factor-1
GDF15 and *Gdf15*: Growth differentiation factor 15
NQO1 and *Nqo1*: NAD(P)H dehydrogenase [quinone] 1
TNFSF4 and *Tnfsf4*: TNF superfamily 4
UBD and *Ubd*: Ubiquitin D
